# Reliability of landmark identification for analysis of the temporomandibular joint in real-time MRI

**DOI:** 10.1186/s13005-024-00411-7

**Published:** 2024-02-17

**Authors:** Jérémy Mouchoux, Philipp Meyer-Marcotty, Florian Sojka, Peter Dechent, Daniela Klenke, Bernhard Wiechens, Anja Quast

**Affiliations:** 1 Poliklinik Für Kieferorthopädie, Universitätmedizin Göttingen (UMG), Göttingen, Germany; 2 Institut Für Kognitive Neurologie, Universitätmedizin Göttingen (UMG), Göttingen, Germany

**Keywords:** Magnetic resonance imaging, Temporomandibular joint, Reliability, Cine magnetic resonance imaging, Quantitative evaluation

## Abstract

**Background:**

Real-time magnetic resonance imaging (rtMRI) is essential for diagnosing and comprehending temporomandibular joint (TMJ) movements. Current methods for tracking and analysis require manual landmark placement on each acquisition frame. Therefore, our study aimed to assess the inter- and intra-rater reliability of placing cephalometric landmarks in frames from a dynamic real-time TMJ MRI.

**Material and methods:**

Four real-time MRIs of the right TMJ were taken during mandibular movement at ten frames per second. Seven dentists identified ten landmarks on two frames (intercuspal position—ICP—and maximum mouth opening—MMO) twice at a two-week interval, yielding 112 tracings. Six typical cephalometric measurements (angles and distances) were derived from these landmarks. The reliabilities of landmarks and measurements were evaluated using distance-based (dbICC), linear mixed effect model intraclass correlation (lmeICC), and standard ICC.

**Results:**

The average inter-rater reliability for the landmarks stood at 0.92 (dbICC) and 0.93 (lmeICC). The intra-rater reliability scores were 0.97 and 0.98. Over 80% of the landmarks showed an ICC greater than 0.98 (inter-rater) and over 0.99 (intra-rater). The lowest landmark ICC was observed for the orbitale and the oblique ridge of the mandibular ramus. However, the cephalometric angle and distance measurements derived from these landmarks showed only moderate to good reliability, whereas the reliability in the frames with ICP was better than those with MMO. Measurements performed in the ICP frame were more reliable than measurements in the MMO frame.

**Conclusion:**

While dentists reliably localize isolated landmarks in real-time MRIs, the cephalometric measurements derived from them remain inconsistent. The better results in ICP than MMO are probably due to a more familiar jaw position. The higher error rate of the TMJ measurements in MMO could be associated with a lack of training in real-time MRI analysis in dentistry.

## Background

A physiological condyle–fossa relationship of the temporomandibular joint (TMJ) is considered an essential factor for the correct function of the stomatognathic system. It therefore plays a major role in dental treatment planning [1, 2]. Deviations of condylar position or shape have been correlated with temporomandibular disorders (TMD), malocclusions, asymmetries, or functional impairments [3–5]. However, many of these findings are based on static imaging examination, disregarding the importance of mandibular movement for condylar function.

Several attempts have been made to add dynamic information to TMJ diagnostics using axiography based on mechanical, optoelectronic, or ultrasound devices [6–8]. However, most of these systems have the limitation that they do not record condylar movement directly but use the movement of the mandibular incisors as a surrogate parameter. Nevertheless, this problem can be solved using real-time magnetic resonance imaging (rtMRI). Static MRI is already increasingly used thanks to its non-ionizing electromagnetic radiation, giving it an edge over Cone Beam Computed Tomography (CBCT) [9]. Since MRI works by detecting the reaction of the water molecules of the tissue rather than by a ray absorption, the organic materials react differently than they do through CBCT or cephalograms. The visualization is, therefore, also different. Research has been done to select better parameters and improve the visualization, especially within the area of the mouth containing metal parts such as screws and braces [10]. On top of it, rtMRI provides a promising approach in dynamic TMJ evaluation and shows good visualization of the condylar disk, enabling individualized measurements and diagnostics [10]. Recently, the development of new techniques improved the 30-year-old dynamic acquisition of the TMJ [11] to observe it more clearly [12] at a very high frequency [13] or through several slices [14]. This may help to receive valuable information to complement static acquisition and to allow a better diagnosis for individual patients [15, 16] without being able to replace the static MRI acquisition as gold-standard for TMD diagnosis [15, 16].

The movement of the mandibular condyle is a complex combination of translation and rotation, with significant variability between individuals [17, 18]. Therefore, most attempts to analyze rtMRI are based on qualitative descriptions of the motion [19, 20]. Nevertheless, to implement a large-scale understanding of the dynamic movement of the complex condyle-disc-fossa-relationship, it is necessary to enable quantitative measurements of the dynamic acquisition following evidence-based medicine principles. Only a few studies implemented such quantitative measurements, describing the movement of several points of the mandibula [21] or the condyle [22], relying on manually placed landmarks frame by frame, notably to compute the instantaneous center of rotation or the rotation and translation of the condyle. However, the results obtained with this procedure are very sensitive to the correct identification of the landmarks [23, 24], which raises the question of how reliable these measurements are. Therefore, this study aims to assess the inter- and intra-rater reliability for localizing cephalometric landmarks in dynamic rtMRI of the TMJ and the cephalometric measurement of distances and angles relying on those landmarks. The increased use of MRI paired with its different tissue visualization might raise the need for new landmarks, better defined for MRI than the standard bone-based landmarks. Three ICCs are used and compared in this study to prepare for this potential arrival. Two based directly on the landmarks, the distance-based intraclass correlation (dbICC) proposed by Xu et al. [25] and the linear mixed effect intraclass correlation (lmeICC) proposed by Chen et al. [26], and one, the standard ICC, applied on the distance measurement defined by the landmarks.

## Methods

### Patients

This study was approved by the Institutional Ethics Committee (no. 6/7/21) following the Declaration of Helsinki. All patients gave written informed consent to participate in the study.

The data for this study were based on four healthy adult patients (mean age: 32 ± 8.5 years; male: *n* = 2, female: *n* = 2). Patients showed natural skeletal configuration (class I), full dentition, and absence of temporomandibular disorder (TMD) symptoms. Exclusion criteria were age below 18, craniofacial anomaly, large tattoos, and intraoral or intracorporal metal components such as orthodontic treatment, pacemaker, cochlear or joint implants…

### Acquisition of rtMRI

Each patient was asked to open and close the mouth within a time interval of 10s during the rtMRI acquisition of the TMJ and the stomatognathic system according to the protocol by Krohn et al. [22]. MRIs were recorded through a Siemens Magneton Prisma fit with T2/T1 contrast (refocused FLASH) at ten frames per second. The in-plane resolution was 0.75 × 0.75 mm for a field of view of 128 × 128mm. The slice thickness was set to 6.0mm, echo time (TE) to 1.56ms, repetition time (TR) to 2.56ms, and the number of excitations (NEX) to 1. After a static calibration scan in sagittal, coronal, and transversal planes, three slices were positioned on the center of the condyle, aligned with the mandibular ramus at its rest position and inclined to include most of it when the mouth was fully open (17.7° ± 4.0° compared to the sagittal plane) with 6mm inter-slice.

Standardized instructions to open and close the mouth were displayed to the patient on an LCD monitor (15″, FHD 1.920 × 1.080) from a DELL Latitude 5520 laptop (Dell, Round Rock, United States) during each session with the following protocol:

- Phase 1: Ten seconds of rest
- Phase 2: Four cycles of maximum mouth opening starting and ending at the intercuspal position, lasting ten seconds each
- Phase 3: Ten seconds of rest.

Both sides of the TMJ were acquired sequentially. However, to avoid patient-dependent variables and keep the number of rated scans low, inter- and intra-rater reliability was evaluated on the right TMJ of each patient. This side was randomly chosen at the start of the study.

### Landmarks and measurements of angles/distances

For the identification of the cephalometric landmarks and the measurements of angles/distances based on those, two frames of the rtMRI were selected to compare every patient in the same position: (1) Intercuspal position (ICP; defined as the last frame of the first rest phase), and (2) maximum mouth opening position (MMO; defined as the frame displaying the maximum movement of the mouth in phase 2). No intermediate frame was included due to the variance in mouth opening between the individuals despite the visual instruction.

**Fig. 1:**
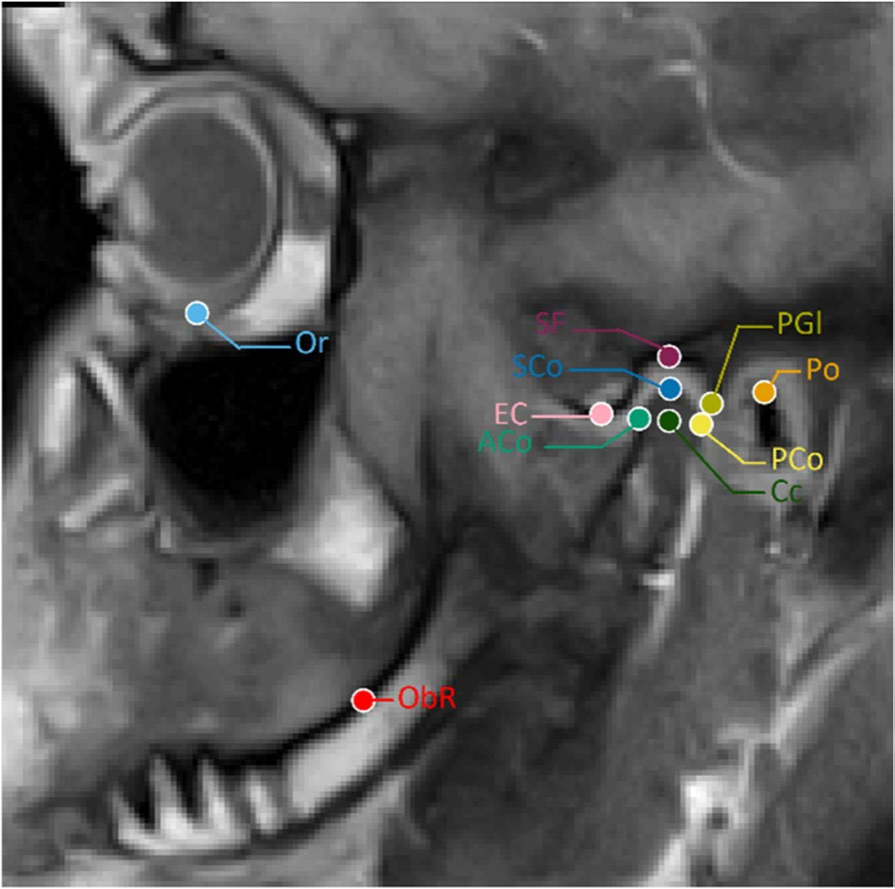
Approximate location of the landmarks: During training sessions, the raters were demonstrated the landmarks at these positions

Each rater was asked to place ten landmarks, commonly used in clinical settings and well-described in the literature, to identify different anatomical structures relevant to the analysis of the stomatognathic system, as illustrated in Fig. 1 and described in Table 1. Based on these landmarks, six common cephalometric angular and linear measurements were calculated, as described in Table 2.

**Table 1:** Description of the cephalometric landmarks

Landmark	Abbreviation	Description
porion	Po	the most superior point of the bony external auditory meatus
orbitale	Or	The most inferior and anterior point of the infraorbital rim
anterior condyle	ACo	the most anterior point of the condyle
posterior condyle	PCo	the most posterior point of the condyle
superior condyle	SCo	the most superior point of the condyle
centre of the condyle	Cc	the central point of the condyle between Aco and PCo
superior fossa	SF	the most superior point of the fossa articularis
eminence crest	EC	the most inferior point of the eminence crest
post-glenoid process	PGI	the most inferior point of the post-glenoid process
oblique ridge of the ramus	ObR	oblique ridge of the ramus at the transition from the corpus to the ramus mandibulae

**Table 2:** Description of the measurements

Measurements	Description
Inclination fossa	Angle in degrees between the EC to SF line and the Frankfurt horizontal plane
Inclination ramus	Angle in degrees between the SCo to ObR line and the Frankfurt horizontal plane
Distance Condyle to FHL	Euclidian distance in mm between the condyle centre and its projection on the Frankfurt horizontal plane
Condylar length	Euclidian distance between ACo and PCo
Superior joint space	Euclidian distance between SCo and SF
Length Fossa	Euclidian distance between SF and EC

### Raters

Seven post-graduated dentists – familiar with cephalometric analysis on lateral X-rays—placed the landmarks on 8 MRI frames (right condyle of 4 patients: 4xICP; 4xMMO; randomized order for each rater) using a custom Matlab [27] application, enabling them to zoom in and out and to set and correct each landmark individually. The screen resolution was 1920 × 1080 for a size of 15 inches. The software determined each landmark’s x- and y-coordinate and calculated the angular/distance measurements. Each dentist rated the scans twice with an interval of two weeks. The first rating session started with two training frames (left condyle of 1 patient: 1xICP; 1xMMO), which were not included in the analysis to prevent the learning curve effect.

### Calculation of inter- and intra-rater reliability

Two methods were applied to assess the reliability of the landmarks:

- (1) The distance-based intraclass correlation (dbICC) proposed by Xu et al. [25]: The dbICC is computed as the fraction of the average within-individual distances on the between-individual distances. Simply said, it is one minus the ratio of distances between the points explained by the grouping (inter- or intra-raters) over all the distances.
- (2) The linear mixed effect intraclass correlation (lmeICC) based on fitting a mixed model on the data: The multi-dimensions are defined as two different variables. lmeICC is then one minus the ratio of variance explained by the grouping over the whole variance. Chen et al. [26] propose four modeling strategies according to the experiment model. As our protocol is an absolute agreement version (ICC(2,1): Two-way random effects, absolute agreement, single measurement) without regularization or measurement error, we applied the Linear mixed effect model (LME) to compute lmeICC with the formula (1) below using Matlab 2019a.
  1 $$\begin{aligned} Coordinate\sim1+Repetition+Dimension\ast Condylar\;Position+\left(1\vert Rater\right)\\+\left(Dimension\ast Condylar\;position\vert Patient\right)\end{aligned}$$

The inter- and intra-rater reliability of the angular/distance measurements were calculated through the standard ICC(2,1) according to the recommendations by Terry et al. [28].

## Results

The inter- and intra-rater reliability of landmark identification and related angular/distance measurements in rtMRI was calculated based on 112 cephalometric tracings (7 raters × 2 TMJ positions × 4 patients × 2 rating sessions).

### Landmarks

**Fig. 2:**
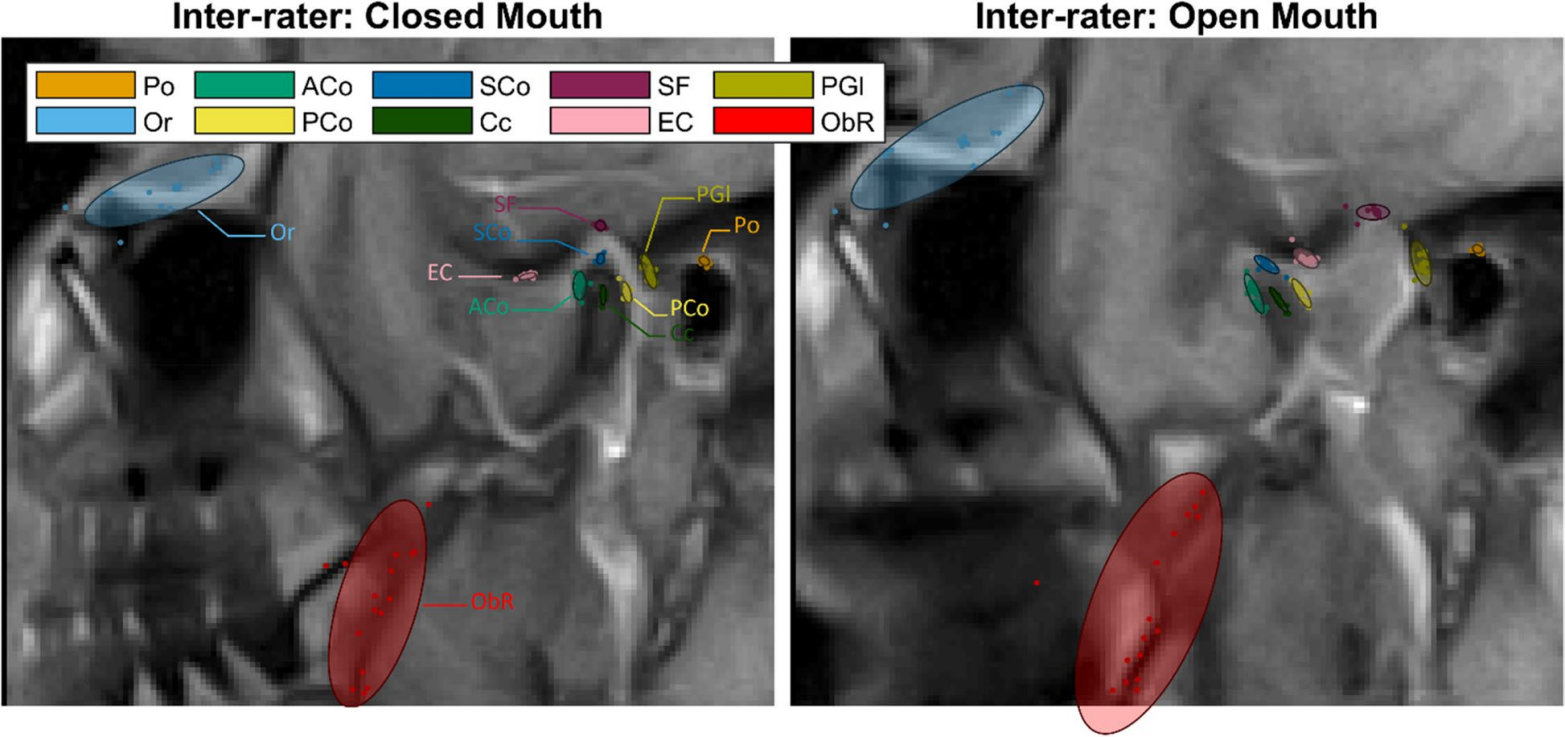
Scan of a patient at the two mouth positions with the landmarks placed by the raters and the ellipse of confidence at 80% for each of the landmarks. The scan has been cropped to the area of interest

The two ICC methods gave similar results for landmark identification. The total mean inter-rater reliability for the landmarks was 0.92 for dbICC and 0.93 for lmeICC. The inter-rater reliability of porion, the anterior condyle, the posterior condyle, the superior condyle, the center of the condyle, the superior fossa, the eminence crest, and the post-glenoid process ranged between 0.98 and 0.99 (see Tables 3 and 4) which is proof of excellent reliability according to the scale given by Koo et al. [28]. The ICC results for the landmarks orbitale and oblique ridge of the ramus indicated only moderate reliability. Figure 2 displays the variance of landmark identification between the raters.

**Table 3:** dbICC Inter-rater, floored to the centile for both positions (Total), the Intercuspal Position (ICP) and the Maximal Mandibular Opening (MMO)

Point	Total	ICP	MMO
Po	0,99	0,99	0,99
Or	0,65	0,74	0,56
ACo	0,99	0,99	0,98
PCo	0,98	0,99	0,98
SCo	0,99	0,99	0,99
Cc	0,99	0,99	0,99
SF	0,99	0,99	0,99
EC	0,99	0,99	0,99
PGl	0,99	0,99	0,99
ObR	0,6	0,63	0,57

**Table 4:** lmeICC Inter-rater, floored to the centile for both positions (Total), the Intercuspal Position (ICP) and the Maximal Mandibular Opening (MMO)

Point	Total	ICP	MMO
Po	0,99	0,99	0,99
Or	0,68	0,73	0,62
ACo	0,98	0,98	0,98
PCo	0,98	0,99	0,98
SCo	0,99	0,99	0,99
Cc	0,99	0,99	0,99
SF	0,99	0,99	0,99
EC	0,99	0,99	0,99
PGl	0,99	0,99	0,98
ObR	0,68	0,67	0,64

The intra-rater reliability was 0.97 for dbICC and 0.98 for lmeICC (see Tables 5 and 6). The median intra-rater reliability of the porion, the anterior condyle, the posterior condyle, the superior condyle, the center of the condyle, the superior fossa, the eminence crest, and the post-glenoid process ranged between 0.97 and 0.99. Again, the poorest ICC values were found for the landmarks orbitale and oblique ridge. However, their intra-rater reliabilities are still rated as good to excellent.

**Table 5:** dbICC Intra-rater values are given as the median (minimum–maximum) of the test–retest of all the raters, floored to the centile for both position (Total), the Intercuspal Position (ICP) and the Maximal Mandibular Opening (MMO)

Point	Total	ICP	MMO
Po	0.99 (0.99—0.99)	0.99 (0.99—0.99)	0.99 (0.99—0.99)
Or	0.94 (0.53—0.99)	0.97 (0.73—0.99)	0.95 (0.33—0.99)
ACo	0.99 (0.98—0.99)	0.99 (0.99—0.99)	0.99 (0.98—0.99)
PCo	0.99 (0.98—0.99)	0.99 (0.98—0.99)	0.98 (0.97—0.99)
SCo	0.99 (0.99—0.99)	0.99 (0.99—0.99)	0.99 (0.99—0.99)
Cc	0.99 (0.99—0.99)	0.99 (0.99—0.99)	0.99 (0.98—0.99)
SF	0.99 (0.99—0.99)	0.99 (0.99—0.99)	0.99 (0.99—0.99)
EC	0.99 (0.99—0.99)	0.99 (0.99—0.99)	0.99 (0.99—0.99)
PGl	0.99 (0.98—0.99)	0.99 (0.98—0.99)	0.99 (0.97—0.99)
ObR	0.84 (0.43—0.91)	0.89 (0.52—0.98)	0.82 (0.35—0.87)

**Table 6:** lmeICC Intra-rater values are given as the median (minimum–maximum) of the test–retest of all the raters, floored to the centile for both position (Total), the Intercuspal Position (ICP) and the Maximal Mandibular Opening (MMO)

Point	Total	ICP	MMO
Po	0.99 (0.99—0.99)	0.99 (0.99—0.99)	0.99 (0.99—0.99)
Or	0.96 (0.53—0.99)	0.96 (0.72—0.99)	0.93 (0.28—0.99)
ACo	0.99 (0.98—0.99)	0.99 (0.99—0.99)	0.99 (0.98—0.99)
PCo	0.99 (0.98—0.99)	0.99 (0.97—0.99)	0.98 (0.97—0.99)
SCo	0.99 (0.99—0.99)	0.99 (0.99—0.99)	0.99 (0.99—0.99)
Cc	0.99 (0.99—0.99)	0.99 (0.99—0.99)	0.99 (0.98—0.99)
SF	0.99 (0.99—0.99)	0.99 (0.99—0.99)	0.99 (0.99—0.99)
EC	0.99 (0.99—0.99)	0.99 (0.99—0.99)	0.99 (0.99—0.99)
PGl	0.99 (0.98—0.99)	0.99 (0.98—0.99)	0.99 (0.97—0.99)
ObR	0.88 (0.70—0.96)	0.89 (0.62—0.98)	0.86 (0.37—0.93)

The lowest observed intra-rater reliability for orbitale was 0.73 for the ICP and 0.33 for the MMO in the dbICC analysis, and 0.72 for the ICP, and 0.28 for the MMO in the lmeICC analysis. The same rater obtained those low values in ICP and MMO. For the oblique ridge of the ramus, the lowest intra-rater reliability was 0.52 for the ICP and 0.35 for the MMO (dbICC), and 0.62 for the ICP and 0.37 for the MMO (lmeICC). Interestingly, those poorest results were obtained from different raters in ICP and MMO.

In conclusion, the ICC of 80% of the landmarks was higher than 0.98 for inter-rater reliability and higher than 0.99 for intra-rater reliability. Landmarks set on frames in ICP revealed higher reliability compared to landmarks set on frames in MMO.

### Angular/distance measurements

All six measurements could be computed based on the landmarks placed by the raters. Overall, the measurements of angles and distances derived from the landmarks demonstrated moderate to good reliability (mean intra-rater ICC: 0.81; mean inter-rater ICC: 0.59, see Table 7). In the ICP frame, the inter-rater reliability ranged from 0.61 for the distance between the condyle and the fossa to 0.76 for the distance from the condyle to the Frankfurt horizontal plane. The measurements in the MMO frame showed reliabilities from 0.11 for the distance from the condyle to the Frankfurt horizontal plane to 0.77 for the distance from the condyle to the fossa. In general, measurements performed in the ICP frame were more reliable than measurements in the MMO frame, except for the distance between the condyle and fossa.

**Table 7:** Inter- and intra- rater reliabilities of the measurements, floored to the centile for both positions (Total), the Intercuspal Position (ICP) and the Maximal Mandibular Opening (MMO). The values for the intra-rater reliabilities are given as median (minimum–maximum)

Point	Inter-rater			Intra-rater		
	**Total**	**ICP**	**MMO**	**Total**	**ICP**	**MMO**
Angle Fossa—FHL	0,59	0,67	0,52	0.86 (0.50—0.96)	0.80 (0.51—0.97)	0.91 (0.30—0.97)
Angle Mandibula	0,53	0,63	0,44	0.71 (0.42—0.92)	0.91 (0.01—0.96)	0.64 (0.31—0.90)
Dist Condyle to FHL	0,44	0,76	0,11	0.86 (0.35—0.97)	0.87 (0.79—0.98)	0.83 (-0.14—0.96)
Length Condyle	0,66	0,72	0,59	0.71 (0.46—0.84)	0.79 (0.66—0.97)	0.72 (0.15—0.88)
Dist Condyle to Fossa	0,69	0,61	0,77	0.85 (0.21—0.92)	0.77 (-0.33—0.94)	0.91 (0.76—0.97)
Length Fossa	0,63	0,63	0,62	0.72 (0.27—0.89)	0.70 (0.03—0.97)	0.71 (0.39—0.90)

The median ICC of the intra-rater reliability was relatively homogeneous, ranging from 0.70 for the length of the fossa to 0.91 for the angle of the mandibula in ICP and from 0.64 for the angle of the mandibula to 0.91 for the distance condyle to fossa in the MMO. However, the minimum ICC showed huge variance with heterogeneous values from -0.33 for the distance between the condyle and the fossa to 0.79 for the distance between the condyle and the Frankfort Horizontal Plane for ICP. Similar results were obtained for MMO.

## Discussion

To the best of our knowledge, this study is the first to measure the inter- and intra-rater reliability for the localization of cephalometric landmarks in dynamic rtMRI of the TMJ and angles/distances relying on those landmarks. Therefore, seven raters identified twice ten landmarks on eight images (4xICP; 4xMMO) of the right condyle. The two ICC methods applied provided similar ICC values. In general, inter- and intra-rater reliability was excellent for landmark localization except for orbitale and the oblique ridge of the ramus. However, corresponding measurements of angles and distances showed only moderate to good reliability caused both by a more severe ICC and by the addition of errors in landmark identification. For instance, it needs four landmarks to calculate the ramus’s inclination: the center of the condyle, the oblique ridge of the ramus, the orbitale, and the porion. Suppose all of these landmarks include a small deviation from the ideal position: this could result in a much greater error in the angle for geometric reasons, depending on the axis of error (which generally follows the contour of the anatomical structure).

As no previous reliability analysis in dynamic rtMRI exists, our results can only be compared to reliability in static MRI. For example, Heil et al. [29] obtained excellent inter- and intra-rater reliabilities for similar measurements with a voxel size of 0.68 × 0.68x0.68mm and 2.2s per slice as well as Juerchott et al. [30] who performed MRI with 0.53 × 0.53x1.1mm per pixel and 1.6s per slices. Four potential causes have been identified to explain the lower reliability obtained in our study based on dynamic rtMRI: the novelty of MRI, the number of raters, and the quality of the dynamic images.

First, MRI acquisitions do not belong to the standards of orthodontic interventions. Both studies based on static acquisition only included two raters who were probably experts in MRI analysis. The raters in our studies were general dentists undergoing postgraduate training in orthodontics with at least two years of experience in radiological examinations. They were well-trained in identifying cephalometric landmarks in lateral cephalograms but had little experience in MRI analysis of the TMJ. During the first rating session, they received special training on MRI images in ICP and MMO. Nevertheless, it must be remembered that MRI displays bony structures in a reverse manner to lateral cephalograms, which might have been confusing for the raters and may contribute to the only moderate reliability of the angular/distance measurements. The inclination of the acquisition plane, which is not parallel to the sagittal plane to contain the condyle and the ramus during the whole movement, also impacts the shape of the anatomical structures observed on the scan. Interestingly, the results in frames with ICP were better than those with MMO, which probably can be attributed to the fact that images in ICP are more familiar to dentists than those in MMO. Even if the acquisition plan was positioned and inclined to follow most of the movement of the ramus, the movement of the jaw is too complex to completely avoid a slight displacement of the jaw normal to the acquisition plane, which implies small changes in the observed shape of the mandibula or superimpositions of structures. The distance between the condyle and the fossa showed the opposite trend because this distance is longer in MMO, which mathematically decreases the ratio between the rating variability and the mean. Increased training sessions or selection of radiologists might improve the ICC results. However, the present study aimed to evaluate the usability of rtMRI images in the daily clinical routine, in which dentists and orthodontists without special training are the target group. A possible future solution could come from the progress of supportive AI in orthodontics. Indeed, the automatic placement of landmarks on cephalograms improved to reach 88.32% of successful detection rate in the range of two millimeters [31]. Automatic condyle segmentation has also improved and now has excellent reliability [32, 33]. To solve the placement of landmarks on rtMRI, we could, therefore, register the lateral cephalogram on the MRI to report the landmarks placed on the standard scan on the MRI in ICP. Afterwards, a new tracking of the mandibula during rtMRI using all its available pixels rather than a few landmarks should be developed. This would enable monitoring the evolution of those landmarks during the movement of the mandibula with more accuracy and reliability and, therefore, would provide their positions at MMO.

Second, studies including more raters usually display higher variance of ICC and lower reliability results. März et al. [34] recruited five raters and obtained only good internal reliability for a setting similar to Heil et al. [29] with ICC coefficients of 0.74 and above (in contrast Heil et al.: ICC > 0.93). The high variance between the raters has also affected the results of our study. One rater especially demonstrated poor intra-rater reliability regarding identifying the orbitale and oblique ridge of the ramus. Automatic landmark identification using artificial intelligence might help to solve this issue [35].

Last, in addition to the human factor, the technical limitations of real-time MRI must be considered. The dynamic character of the MRI affects image quality. The dynamic acquisition, which acquires slices every 0.1 s, can produce a blurred vision of the anatomical structure due to the movement and relatively thick slices. Apart from this blurredness in the moving areas, no motion-related artifacts could be observed. Moreover, the rtMRI acquisition and the selected frames at ICP and MMO focused on good visualization of the TMJ and its motion. Accordingly, landmarks in the TMJ region received excellent ICC values. More distant landmarks like orbitale and oblique ridge of the ramus performed worse. This might be caused by projection errors or impaired depiction of these areas. A combination of high spatial resolution static and high temporal resolution dynamic MRI and specific postprocessing software might help overcome both problems in the future.

## Conclusion

First, as the usage of MRI is growing in dentistry and orthodontics, new landmarks might be created to replace some standard bone-defined landmarks that are invisible on the MRI scans. Our results show that the 2D methods currently present in the literature to evaluate reliability are much more laxist than the standard ICC applied to the measurements based on landmarks. As it is those measurements that are of interest to the experimenter, the reliability of a specific landmark should always be studied through the reliability of the measurements based on it instead of the landmark reliability directly.

Second, the present study investigated inter- and intra-rater reliability with the rationale that landmarks were previously used to track and quantify the rotation and the translation of the condyle and the mandible on dynamic rtMRI [21, 22]. Implementing this approach in the clinical routine requires landmark identification to be reliable throughout different raters. As demonstrated by our data, the reliability shows high variability between raters and landmarks. Therefore, future attempts should leave manual landmark-based tracking and focus instead on A.I.-assisted pixels-based tracking, a promising approach to provide objective and reliable tracking of the condyle and mandible coming soon.

## Data Availability

The datasets used and analyzed during the current study are available from the corresponding author on reasonable request.

## Abbreviations

CBCT: Cone-beam computer tomography
dbICC: Distance-based intraclass correlation
ICC: Intraclass correlation
ICP: Intercuspal position
lmeICC: Linear mixed effect model intraclass correlation
MMO: Maximal mouth opening
MRI: Magnetic resonance imaging
rtMRI: Real-time magnetic resonance imaging
TMD: Temporomandibular disorders
TMJ: Temporomandibular joint
